# Amoxicillin and thiamphenicol treatments may influence the co-selection of resistance genes in the chicken gut microbiota

**DOI:** 10.1038/s41598-022-24927-7

**Published:** 2022-11-27

**Authors:** Andrea Laconi, Roberta Tolosi, Lapo Mughini-Gras, Matteo Cuccato, Francesca Tiziana Cannizzo, Alessandra Piccirillo

**Affiliations:** 1 Department of Comparative Biomedicine and Food Science, University of Padua, 35020 Legnaro, PD Italy; 2 Institute for Risk Assessment Sciences (IRAS), Faculty of Veterinary Medicine, Utrecht University, Utrecht, The Netherlands; 3 Centre for Infectious Disease Control, National Institute for Public Health and the Environment (RIVM), Bilthoven, The Netherlands; 4 Department of Veterinary Science, University of Turin, Turin, Italy

**Keywords:** Antibiotics, Antimicrobial resistance, Microbiome, Bacterial genes, Next-generation sequencing, Mobile elements, Microbial ecology, Population dynamics

## Abstract

The aim of this study was to assess the dynamics of microbial communities and antimicrobial resistance genes (ARGs) in the chicken gut following amoxicillin and thiamphenicol treatments and potential co-selection of ARGs. To this purpose, the microbial community composition, using 16S rRNA NGS, and the abundance of ARGs conferring resistance to β-lactams and phenicols, using qPCRs, were determined. Results revealed that the administered antimicrobials did not significantly reduce the gut microbiota diversity, but changed its composition, with taxa (e.g. *Gallibacterium* and *Megamonas*) being enriched after treatment and replacing other bacteria (e.g. *Streptococcus* and *Bifidobacterium*). Positive correlations were found between ARGs (e.g. *cmlA*, *bla*_*CMY-2*_, and *bla*_*SHV*_) and the relative abundance of specific taxa (e.g. *Lactobacillus* and *Subdoligranulum*). The selective pressure exerted by both amoxicillin and thiamphenicol resulted in an increased abundance of ARGs conferring resistance to β-lactams (e.g. *bla*_*TEM-1*_, *bla*_*SHV*,_ and *bla*_*CTX-M1-like*_) and phenicols (e.g. *floR* and *cmlA*). These findings, together with the co-occurrence of genes conferring resistance to the two antimicrobial classes (e.g. *bla*_*TEM-1*_ and *cmlA*), suggest a possible interaction among antimicrobials on resistance emergence, possibly due to the presence of mobile genetic elements (MGEs) carrying multiple resistance determinants.

## Introduction

A growing body of evidence suggests that antimicrobial use (AMU) in conventional animal farming contributes to the emergence and spread of antimicrobial resistance (AMR) in bacterial populations, posing a significant threat to both animal and human health^1–3^. Mass drug administration, including antimicrobial administration through feed and drinking water to large numbers of animals at once, is likely to have contributed to the reduced therapeutic efficacy of some antimicrobials and generation of multidrug-resistant bacteria^4^. Chickens harbour bacteria, including human pathogens, resistant to several classes of antimicrobials^5,6^, some of which are listed as critically important antimicrobials by the World Health Organisation (WHO)^7^. Amoxicillin, which belongs to the class of β-lactams, represents one of the most commonly used antimicrobials in humans^8^ and broilers^9^. Previous studies showed that β-lactam antibiotic use can favour the emergence of bacteria resistant to third-generation cephalosporins (3GCs), which represent one of the few available options for treating multidrug-resistant bacterial infections in humans^10^. Thiamphenicol is an analogue of chloramphenicol and a broad-spectrum bacteriostatic antimicrobial, active against both Gram-positive and Gram-negative bacteria^11^. Due to the paucity of new drugs to counteract AMR, dismissed antimicrobials like chloramphenicol, which use is not allowed in animals, regained utility for the treatment of multidrug-resistant bacteria in human medicine^12^. Questions remain as to whether the selective pressure exerted by thiamphenicol might result in the emergence of bacteria resistant to chloramphenicol. ARGs against β-lactams and phenicols can be harboured in mobile genetic elements (MGEs), such as plasmids, and intra- and inter-species (e.g. between *Enterobacteriaceae* and *Clostridiaceae*) horizontal gene transfer (HGT) is a common event contributing to the emergence and dissemination of resistance in microbial communities^13^.

In this study, we assessed whether the prophylactic administration of amoxicillin and thiamphenicol affects the chicken gut microbiota composition, increasing or decreasing the microbial diversity and abundance of specific taxa, as well as whether influences the abundance of ARGs conferring resistance to critically or highly important antimicrobials, such as 3GCs, carbapenems and chloramphenicol. Furthermore, we investigated whether the abundance of specific taxa correlated with ARG occurrence and whether the administration of amoxicillin and thiamphenicol resulted in co-selection of ARGs conferring resistance to the other antimicrobial class.

## Results

### General description of sequences

After the quality filtering step, removal of chimeric fragments, and read merging, a total of 3,378,323 reads with 3007 different features was obtained, with an average of 27,244 sequences per individual sample. After quality filtering, none of the samples was excluded from the analysis of microbial communities.

### Amoxicillin and thiamphenicol treatments influence microbial diversity and the abundance of specific taxa

**Figure 1:**
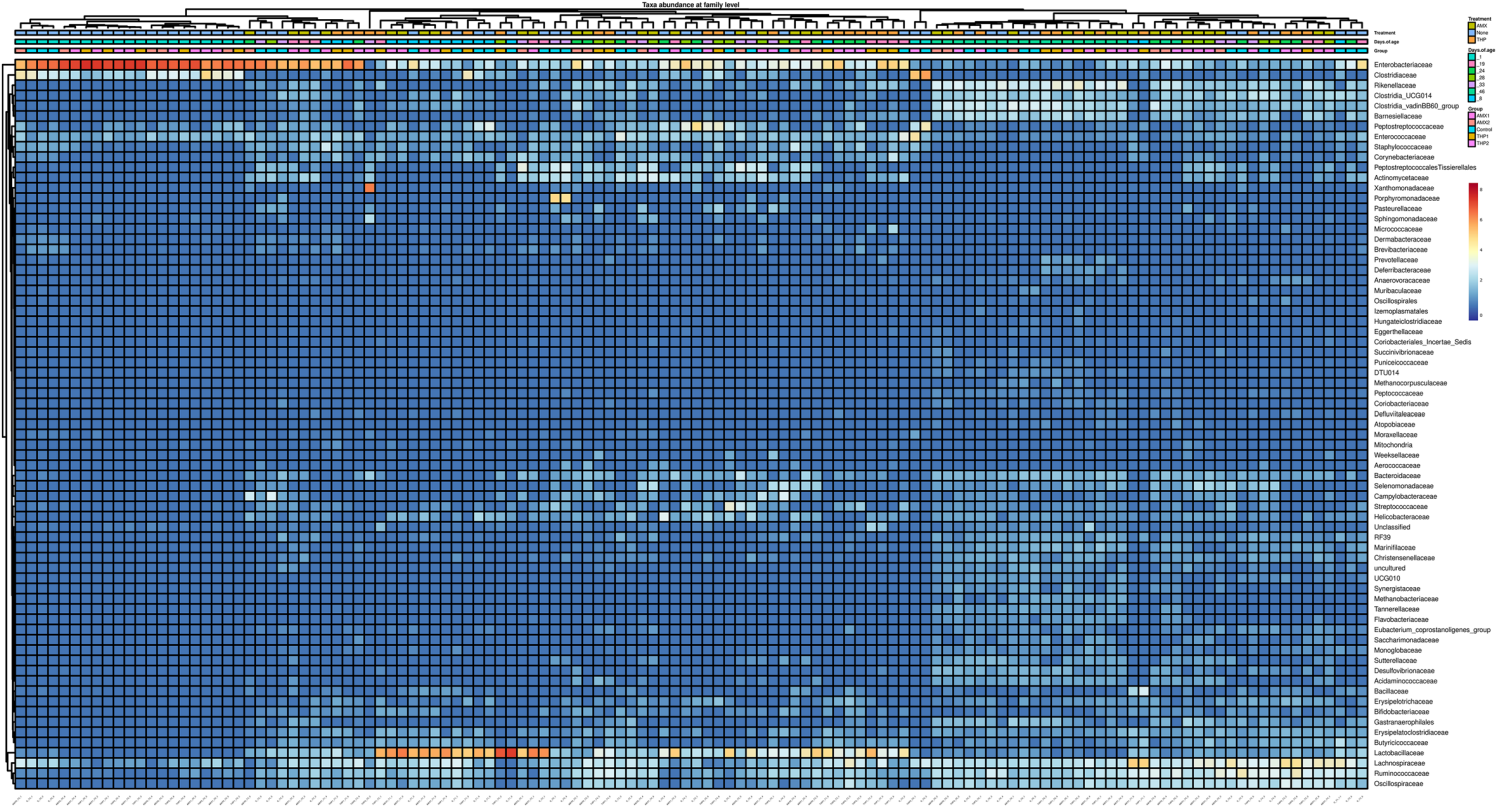
Heatmap representing the microbial community composition at family level. The heatmap was generated in R (version 4.2.1) (https://www.r-project.org/) using package pheatmap (version 1.0.12).

Using 16S rRNA NGS, the gut microbial community composition of the chicks in each group was characterized at different time points. At phylum level, microbiota composition varied with age rather than with treatment (Supplementary Fig. S1). Proteobacteria were the most abundant phyla at 1 day of age (d.o.a.), Firmicutes became dominant at later stages, while Bacteroidota were highly abundant in caecum samples collected at 46 d.o.a. Similar dynamics were observed also at family level, since *Enterobacteriaceae* and *Clostridiaceae* were significantly more abundant at 1 d.o.a. in all groups, *Lactobacillaceae, Lachnospiraceae,* and *Ruminococcaceae* seemed to bloom at 8 d.o.a., and *Rikenellaceae* were the dominant family in the caecum samples collected at 46 d.o.a. (Fig. 1; Supplementary Fig. S2).

#### Early-age administration

**Figure 2:**
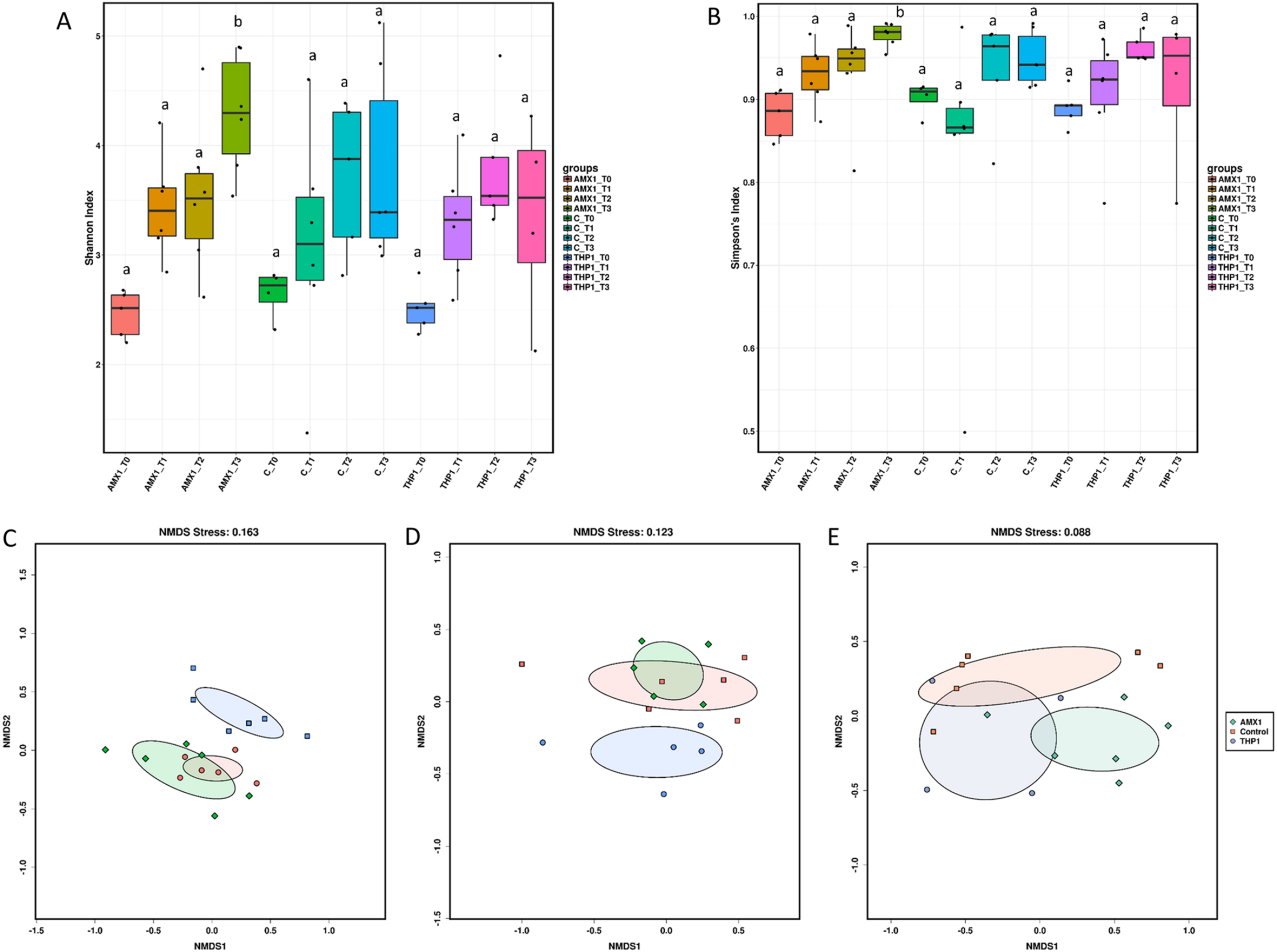
α-Diversity within groups treated at early-age at different time points using Shannon (**A**) and Simpson (**B**) indexes. Boxplots represent 25th to 75th percentiles and whiskers showing a maximum of ×1.5 the interquartile range (IQR), and different letters indicate significant differences within the α-diversity indexes (p < 0.05). β-diversity between treatment groups on day 1 p.t. (**C**), day 12 p.t. (**D**) and day 21 p.t. (**E**). Samples are clustered according to Bray Curtis distances.

In both α-diversity indices (Fig. 2A,B), there was a trend towards increasing diversity from early to late time points in all groups; however, the only significant differences were between the group treated with amoxicillin (AMX1) and the other groups on day 21 post treatment (p.t.), and within AMX1 group between day 21 p.t. and the other time points. PERMANOVA showed that the microbial community was significantly different between the group treated with thiamphenicol (THP1) and the other two groups (i.e. AMX1 and control) on day 1 p.t. (p < 0.001) and on day 12p.t. (p = 0.048), while there were no differences at the last time point. These findings are supported by the NMDS plots (Fig. 2C–E), in which a clear spatial separation between THP1 and the remaining groups at the first two time points was observed.

LEfSe analysis performed at the genus level at each time point showed an increased abundance of genus cc_115 after thiamphenicol administration (LDA = 4.40), and reduction of *Helicobacter* (LDA = 4.40) and *Candidatus Arthomitus* (LDA = 4.49) in the control group on day 1 p.t. (Supplementary Fig. 3A). At the following time point (12 d.p.t.), *Bacteroides* (LDA = 4.79) were reduced in the AMX1 group, while the genus *Streptococcus* was enriched in the control group (Supplementary Fig. 3B). On day 21 p.t. (Supplementary Fig. 3C), six taxa were enriched in the AMX1 group, including *Sphingomonas* (LDA = 4.11), *Megamonas* (LDA = 4.69) and *Bacteroides* (LDA = 4.31), one in the THP1 group, i.e. *Gallibacterium* (LDA = 4.35), and two in the control group, i.e. *Streptococcus* (LDA = 4.65) and *Bifidobacterium* (LDA = 4.13).

#### Middle-age administration

**Figure 3:**
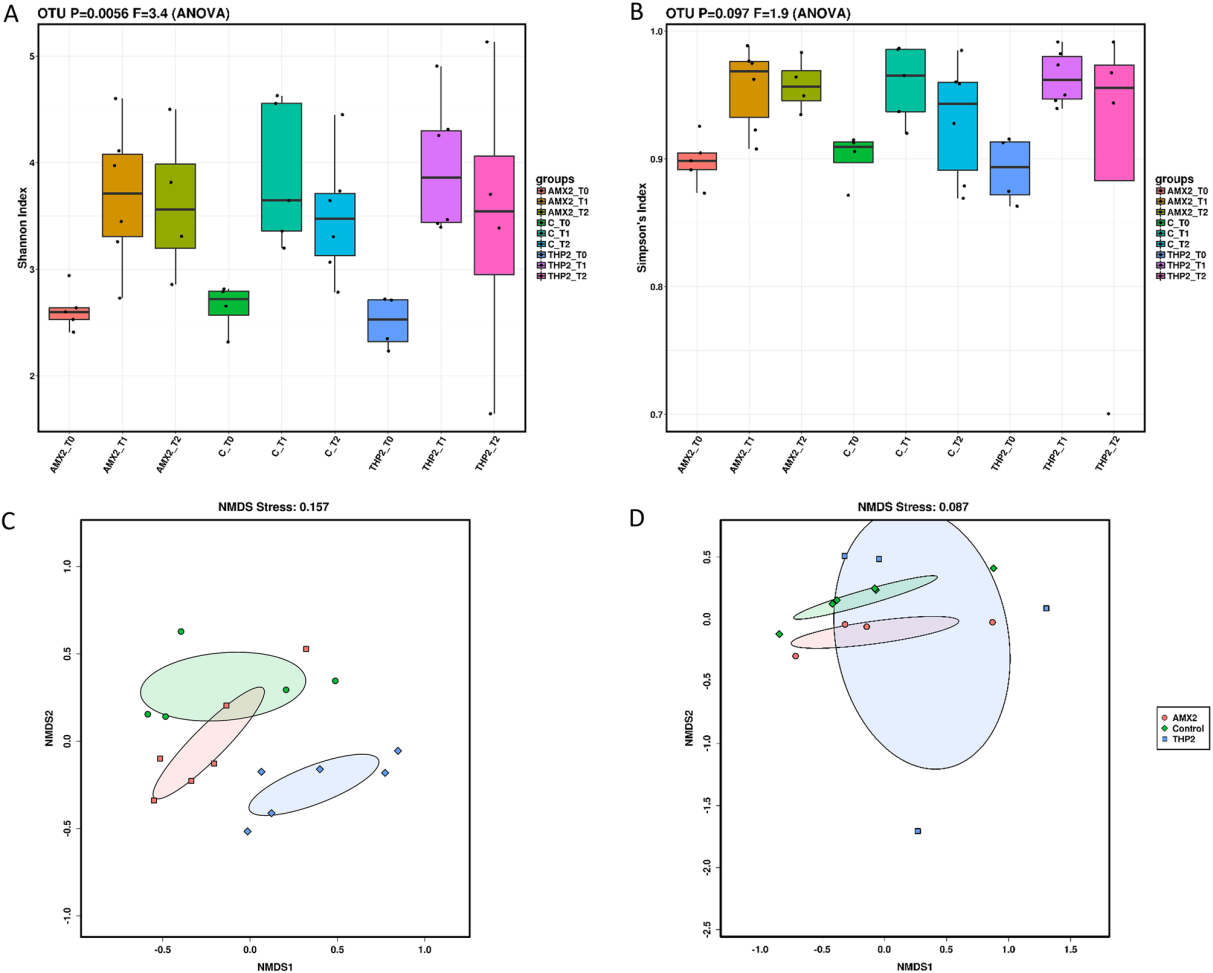
α-Diversity within groups treated at middle-age at different time points using Shannon (**A**) and Simpson (**B**) indexes. Boxplots represent 25th to 75th percentiles and whiskers showing a maximum of ×1.5 the interquartile range (IQR). β-Diversity between treatment groups on day 1 p.t. (**C**) and day 9 p.t. (**D**). Samples are clustered according to Bray Curtis distances.

Middle-age administration of amoxicillin and thiamphenicol did not affect the α-diversity of the gut microbiota, as shown by both Shannon’s and Simpson’s indices (Fig. 3A,B). On the contrary, PERMANOVA and NMDS plot (Fig. 3C,D) showed differences in the microbial community composition (β-diversity) between the group treated with thiamphenicol (THP2) and the other two groups (i.e. amoxicillin treated (AMX2) and control), but only on day 1 p.t. (p < 0.001).

On day 1 p.t., *Megamonas* (LDA = 4.63) was enriched in the AMX2 group, *Ruminococcus* (LDA = 4.61) and cc_115 (LDA = 4.38) in THP2 group, whereas *Helicobacter* (LDA = 4.69) and *Peptoniphilus* (LDA = 4.52) were more abundant in the control (Supplementary Fig. 4A). At day 9 p.t. *Gallibacterium* was more abundant in the thiamphenicol treated group, while *Peptoniphilus* was less abundant in the control group (Supplementary Fig. 4B).

### Amoxicillin and thiamphenicol influence the abundance of β-lactam and phenicol resistance genes

With the exception of *bla*_*NDM*_, all the other ARGs investigated were detected in at least one sample. All of the detected ARGs were also identified in the control group, with the exception of *bla*_*VIM-2*_ and *bla*_*OXA-1*_. Genes conferring resistance to either β-lactams (i.e. *bla*_*TEM-1*_, *bla*_*SHV*_, *bla*_*OXA-1*_ and *bla*_*OXA-48*_) or phenicols (i.e. *catA1*, *catB3*, *floR*, and *cmlA*) were detected in chicks of 1 d.o.a. (Supplementary Fig. S5). In the caecum samples collected at the slaughter house, seven out of fourteen ARGs were identified (i.e. *bla*_*TEM-1*_, *bla*_*CMY-2*_, *catA1*, *catA2*, *catB3*, *floR*, and *cmlA*). All samples were characterized by at least one ARG (min = 1, max = 7, and mean = 3.83), and 89.84% of the samples showed resistance to both antimicrobial classes. Overall, the relative abundance of ARGs conferring resistance to β-lactams and phenicols was significantly increased after treatments; however, genes conferring resistance to β-lactams and phenicols showed different temporal fluctuations in the different groups.

#### Early-age treatment

**Figure 4:**
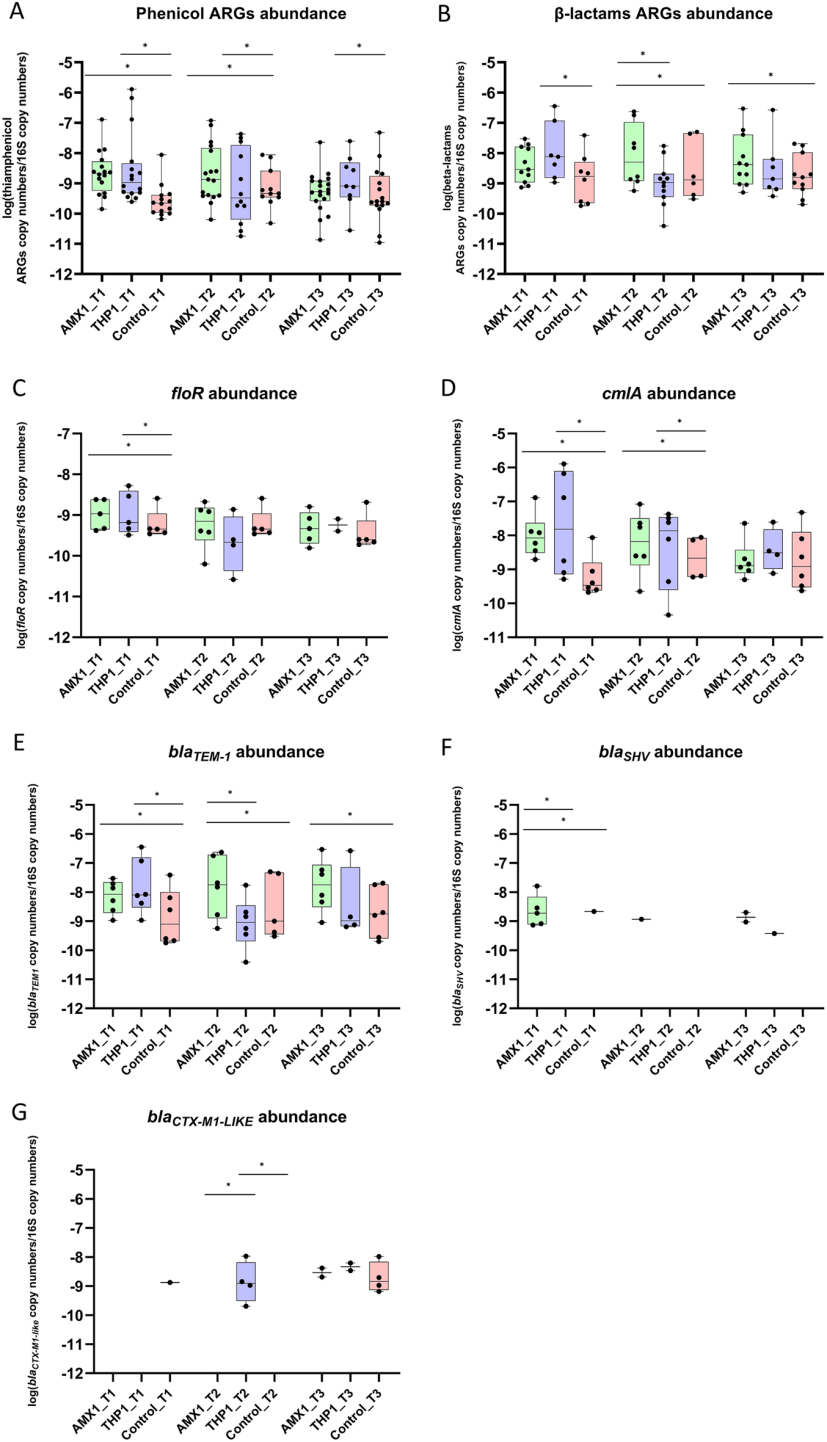
Relative abundance of all genes conferring resistance to β-lactams (**A**) and phenicols (**B**) to 16S rRNA copy number per group per time point after early-age administration of amoxicillin and thiamphenicol. Relative abundance of genes *floR* (**C**), *cmlA* (**D**), *bla*_*TEM-1*_ (**E**), *bla*_*SHV*_ (**F**), and *bla*_*CTX-M1-LIKE*_ (**G**) to 16S rRNA copy number per group per time point. p < 0.05 shown as *. For easiness of representation, only ARGs showing significant differences between groups at the same time point are reported.

The relative abundance of ARGs conferring resistance to phenicols was significantly increased in both AMX1 and THP1 groups compared to the control (Fig. 4A,B); however, while abundance was significantly higher at all time points in the THP1 group, it decreased on day 21 p.t. in the AMX1 group. Compared to the control group, ARGs against β-lactams were more abundant in the THP1 group only on day 1 p.t., while the relative abundance of these ARGs was higher in the AMX1 from day 12 p.t. onwards.

When considering individual ARGs, *floR* was more abundant in both AMX1 and THP1 groups than in the control group only on day 1 p.t. (Fig. 4C), while *cmlA* showed higher abundance in both AMX1 and THP1 groups until day 12 p.t. (Fig. 4D). None of the four remaining ARGs conferring resistance to phenicols was enriched after amoxicillin or thiamphenicol administration at any time point. On day 1 p.t., *bla*_*TEM-1*_ was more abundant in both the AMX1 and THP1 groups compared to the control; however, while the abundance in the AMX1 group remained significantly higher at the following time points, it decreased in the THP1 group (Fig. 4E). *bla*_*SHV*_ was enriched in the AMX1 group on day 1 p.t. (Fig. 4F), while *bla*_*CTX-M1-like*_ was significantly more abundant in the THP1 group compared to the other two groups on day 12 p.t. (Fig. 4G). Of the remaining ARGs conferring resistance to β-lactams, none was enriched after both antimicrobial administrations.

#### Middle-age administration

**Figure 5:**
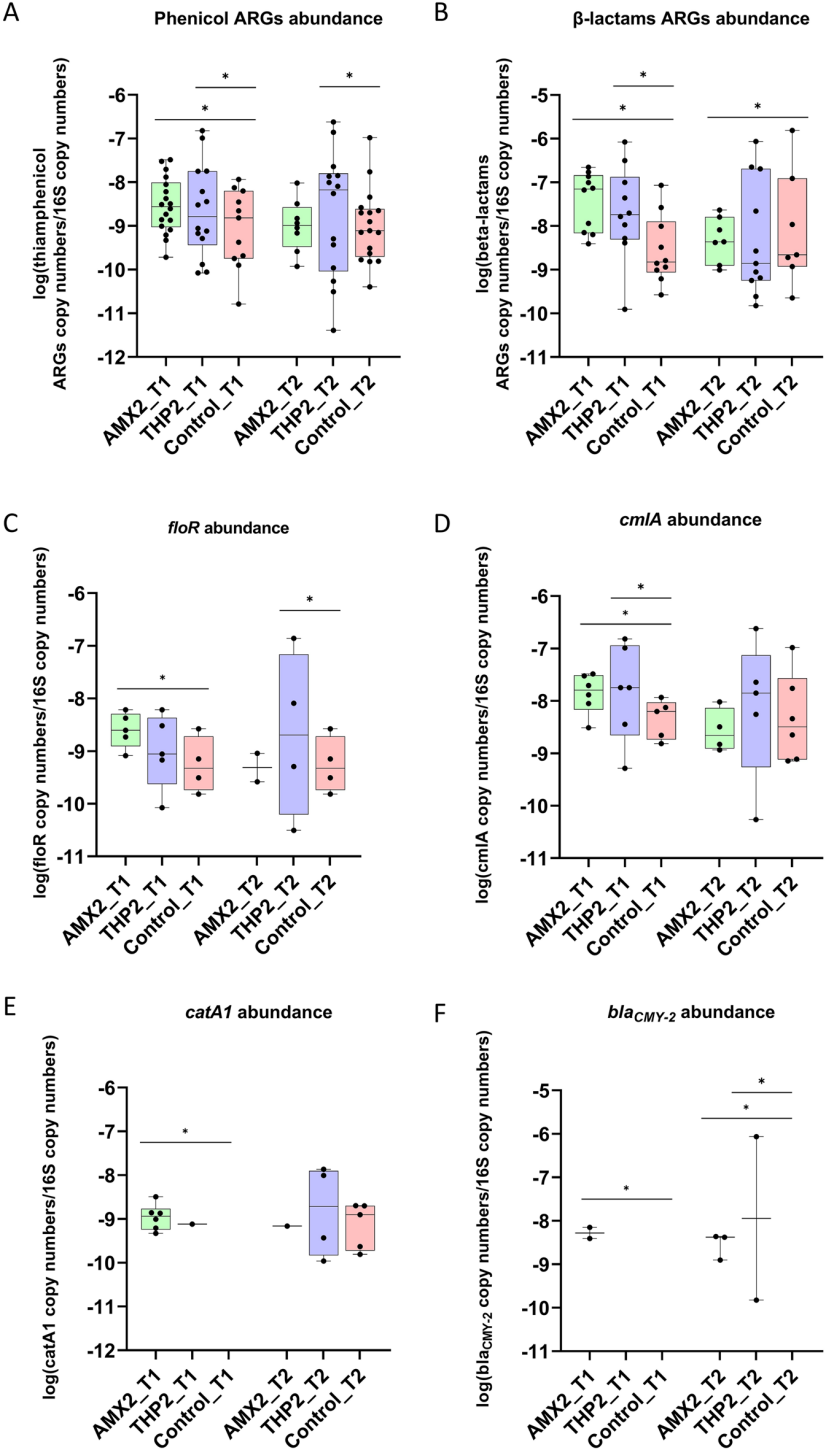
Relative abundance of all genes conferring resistance to β-lactams (**A**) and phenicols (**B**) to 16S rRNA copy number per group per time point after middle-age administration of amoxicillin and thiamphenicol. Relative abundance of genes *catA1* (**C**), *floR* (**D**), *cmlA* (**E**), and *bla*_*CMY-2*_ (**F**), to 16S rRNA copy number per group per time point. p < 0.05 shown as *. For easiness of representation, only ARGs showing significant differences between groups at the same time point are reported.

The relative abundance of both β-lactam and phenicol ARGs was significantly increased in the treated groups on day 1 p.t.; however, on day 9 p.t. only genes conferring resistance to the class of the antimicrobial administered were enriched (Fig. 5A,B). *floR* and *cmlA* were more abundant after the administration of both AMDs but, while *cmlA* was enriched on day 1 post both treatments, *floR* was enriched on day 1 p.t. in the AMX2 group and on day 9 p.t. in the THP2 group (Fig. 5C,D). Interestingly, *catA1*, conferring resistance to phenicols, was enriched only after the administration of amoxicillin (1 d.p.t.), while *bla*_*CMY-2*_, conferring resistance to β -lactams, was enriched in both the AMX2 (1 and 9 d.p.t.) and THP2 (9 d.p.t.) groups (Fig. 5E,F). None of the remaining ARGs was significantly enriched after either treatments at any time-point.

### Microbiome and ARGs composition of caecum samples

**Figure 6:**
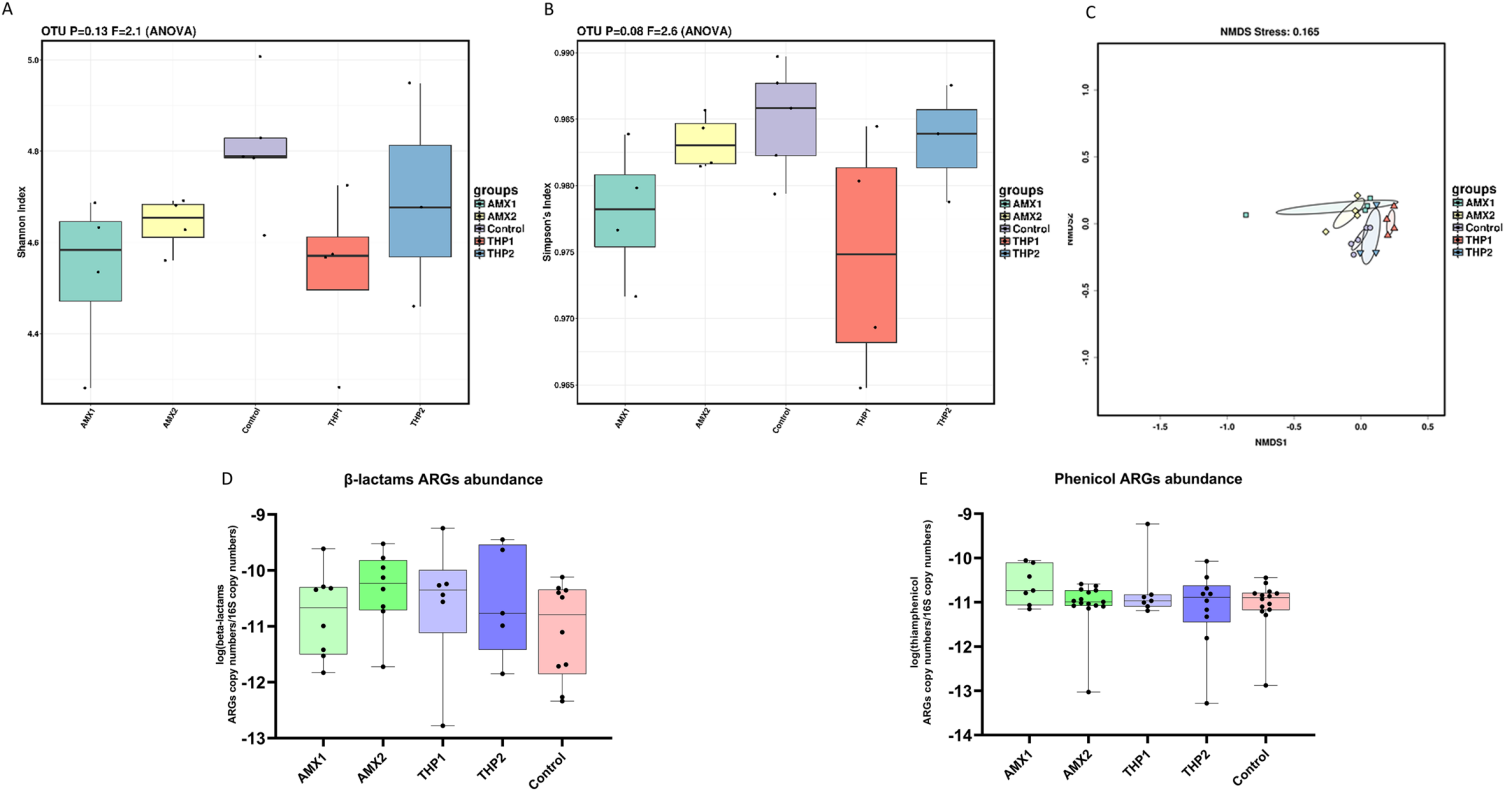
α-Diversity within caecum samples using Shannon (**A**) and Simpson (**B**) indexes. Boxplots represent 25th to 75th percentiles and whiskers showing a maximum of ×1.5 the interquartile range (IQR). β-Diversity between groups (**C**). Samples are clustered according to Bray Curtis distances. Relative abundance of all genes conferring resistance to β-lactams (**D**) and phenicols (**E**) to 16S rRNA copy number per group.

Neither the type of AMDs administered nor the age of treatment seemed to have affected the microbiota diversity of the caecum samples collected at the slaughterhouse, since no significant differences in α- or β-diversity were detected (Fig. 6A–C). Similarly, there were no significant differences in the abundance of ARGs between treated and control groups (Fig. 6D,E).

### Associations between microbial communities and ARGs and co-occurrence of ARGs

The associations between ARGs occurrence and specific taxa (genus level) are summarized in Table 1. Seventeen taxa were significantly associated with at least one ARG, *Lactobacillus* showing the highest number of associations (n = 3), while *cmlA* and *bla*_*CMY-2*_ showed the highest number of associations with specific taxa. The occurrence of *bla*_*CMY-2*_ showed positive associations with five genera (e.g. *Subdoligranulum* and *Butyricicoccus*) and negative associations with *Streptococcus, Lactobacillus*, and *Enterococcus,* while *bla*_*SHV*_ was positively correlated with five taxa, including *Lactobacillus* and *Faecalibacterium*. *cmlA* was positively associated with genera *Lactobacillus*, *Bacteroides*, and *Subdolingranum*, while negative associations were identified with eight taxa, including *Escherichia*/*Shigella* and *Alistipes*. Positive associations were also identified between *floR* and genera *Escherichia*/*Shigella* and *Enterococcus*. Both *catA1* and *catB3* were correlated with an increased abundance of *Ruminococcus torques*, while the latter gene was also associated with *Clostridia*.

**Table 1:** Significant associations between microbial taxa and antimicrobial resistance genes. Z and p values are reported.

Taxa	ARG	z	p
*Escherichia/shigella*	*cmlA*	− 3.04	0.002
	*floR*	2.49	0.013
*Enterococcus*	*bla*_*CMY-2*_	− 2.53	0.011
	*floR*	2.73	0.006
*Unclassified*	*cmlA*	− 3.16	0.002
	*bla*_*CMY-2*_	2.41	0.016
*Lactobacillus*	*cmlA*	3.43	0.001
	*bla*_*SHV*_	2.81	0.005
	*bla*_*CMY-2*_	− 2.24	0.025
*Uncultured*	*cmlA*	− 2.68	0.007
	*bla*_*CMY-2*_	2.43	0.015
*Faecalibacterium*	*cmlA*	− 3.05	0.002
	*bla*_*SHV*_	2.88	0.004
*Erysipelatoclostridium*	*bla*_*SHV*_	4.00	< 0.001
*Ruminococcus_torques_group*	*catA1*	2.08	0.038
	*catB3*	2.31	0.021
*Bacteroides*	*cmlA*	2.88	0.004
	*bla*_*SHV*_	− 6.21	< 0.001
*Incertae Sedis*	*cmlA*	− 2.29	0.022
	*bla*_*SHV*_	2.48	0.013
*Subdoligranulum*	*cmlA*	3.33	0.001
	*bla*_*CMY-2*_	2.80	0.005
*Alistipes*	*cmlA*	− 3.00	0.003
	*bla*_*CMY-2*_	2.73	0.006
*Lachnoclostridiuma*	*floR*	− 3.20	0.001
	*cmlA*	− 2.83	0.005
*Clostridia UCG014*	*cmlA*	− 2.37	0.018
	*catB3*	2.10	0.036
*Butyricicoccus*	*bla*_*CMY-2*_	2.00	0.045
	*bla*_*SHV*_	2.80	0.005
*Streptococcus*	*bla*_*CMY-2*_	− 2.25	0.024
*Staphylococcus*	*bla*_*CTXM1-like*_	− 2.46	0.014

Positive correlations were observed between genes conferring resistance to the same antimicrobial class and also between genes conferring resistance to β-lactams and phenicols. In detail, positive correlations were identified between *bla*_*CMY-2*_ and *catA2* (Spearman r = 0.2561, p = 0.003), between *bla*_*TEM-1*_ and *floR* (Spearman r = 0.3662, p < 0.001) and *cmlA* (Spearman r = 0.4133, p < 0.001), and between *bla*_*CTX-M1-LIKE*_ and *cmlA* (Spearman r = 0.2286, p = 0.009). Data regarding the co-occurrence of ARGs are reported in Table 2.

**Table 2:** Co-occurrence of antimicrobial resistance genes. Spearman’s r and p-values are reported.

	*bla*_*TEM-1*_		*bla*_*CMY-2*_		*bla*_*CTX-M1-LIKE*_		*bla*_*CMY-2*_		*bla*_*VIM2*_		*florR*		*cmlA*		*catA1*		*catA2*	
	p-values	r	p-values	r	p-values	r	p-values	r	p-values	r	p-values	r	p-values	r	p-values	rr	p-values	r
*bla*_*TEM-1*_	–	–	–	–	–	–	< 0.0001	− 0.3734	–	–	< 0.0001	0.3662	< 0.0001	0.4133	–	–	0.023	− 0.2005
*bla*_*CMY-2*_	< 0.0001	− 0.3734	–	–	–	–	–	–	–	–	–	–	0.0251	− 0.1981	–	–	0.0035	0.2561
*bla*_*CTX-M1-LIKE*_	–	–	–	–	–	–	–	–	0.0153	0.2138	–	–	0.0094	0.2286	–	–	–	–
*bla*_*VIM2*_	–	–	–	–	0.0153	0.2138	–	–	–	–	–	–	–	–	–	–	–	–
*florR*	< 0.0001	0.3662	–	–	–	–	–	–	–	–	–	–	< 0.0001	0.5185	< 0.0001	0.3931	–	–
*cmlA*	< 0.0001	0.4133	0.0250	− 0.1981	0.009	0.2286	–	–	–	–	< 0.0001	0.5185	–	–	< 0.0001	0.3511	–	–
*catA1*	–	–	–	–	–	–	–	–	–	–	< 0.0001	0.3931	< 0.0001	0.3510	–	–	–	–
*catA2*	0.0232	− 0.2005	0.0035	0.2561	–	–	–	–	–	–	–	–	–	–	–	–	–	–

## Discussion

The present study investigated longitudinally the selective pressure exerted by the administration of amoxicillin and thiamphenicol on the chicken gut microbiota and associated ARGs. Microbial community showed a strong age-dependent dynamic and, in accordance with previous observations^14,15^, *Enterobacteriaceae* were the predominant taxa at 1 d.o.a., while *Lactobacillaceae, Lachnospiraceae,* and *Ruminococcaceae* dominated the microbiota at later time points. According to previous observations, *Rikenellaceae* were highly abundant in caecal samples collected at 46 d.o.a.^16^. Previous studies reported contrasting effects of AMU on chicken gut microbial diversity (α-diversity). Although Le Roy et al. described that AMU significantly decreased the diversity and richness of the chicken gut microbiota^17^, other studies did not find any significant change, if not a trend toward increased microbial diversity^14,18^. Indeed, the administration of amoxicillin and thiamphenicol does not seem to reduce microbial diversity and richness, to the point that a significant increase in taxonomic diversity was observed in the chicken gut on day 21 after early-age administration of amoxicillin. However, comparison among bacterial communities (β-diversity) revealed that the gut microbiota of chickens treated with thiamphenicol was significantly different from those of the other groups and that early-age administration caused a longer shift in the microbial community composition (i.e. up to 12 d.p.t. in early-age treated group and only on 1 d.p.t. in middle-age treated group). Overall these findings seem to suggest that resistant bacteria could bloom under the selective pressure of amoxicillin and thiamphenicol, compensating for the loss of non-resistant species and that AMD administration at early-age can cause a more persistent perturbation of the gut microbiota. In addition to the risk of selecting for resistant bacterial populations, even if high genetic diversity of the gut microbiota is generally considered beneficial for chicken health, proliferation of usually lowly abundant bacteria might result in an altered microbial community structure, causing metabolic dysfunction and increasing susceptibility to opportunistic pathogens^14,17^.

The relative abundance of ARGs conferring resistance to β-lactams and phenicols increased after administration of either amoxicillin or thiamphenicol compared to the control group. Even if the selection of ARGs against the other antimicrobial class seems to be transitory (i.e. up to 12 d.p.t. in the early-age treated groups and only on 1 d.p.t. in the middle-age treated groups), this finding suggests a potential co-selection for resistance to amoxicillin and thiamphenicol, which is consistent with previous studies reporting increased resistance against β-lactams and phenicols after exposure to florfenicol and amoxicillin, respectively^9,19^. The co-selection and persistence of genes conferring resistance to phenicols (e.g. *cmlA* and *floR*) after exposure to amoxicillin might represent a concern for public health, since in recent years chloramphenicol has been used for the treatment of multidrug-resistant bacteria in humans, especially in low income countries where therapeutic alternatives are scarcely available^12^. Since *bla*_*TEM-1*_, *cmlA*, and *floR* were positively correlated and showed similar dynamics in the chicken gut (i.e. increased abundance after both treatments), the observed co-selection of resistance might rely on the presence of resistance determinants to β-lactams and phenicols on the same genetic element, as previously reported^20,21^. The co-selection for resistance to phenicols and β-lactams seems to involve also AmpC- and extended-spectrum β lactamases (ESBL)-encoding genes, which confer resistance to third-generation cephalosporins (3GCs). Indeed, treatment with thiamphenicol seems to exert a selective pressure on *bla*_*CMY-2*_ and *bla*_*CTX-M1-like*_, which encode for AmpC and ESBL, respectively. Notably, both *bla*_*CMY-2*_ and *bla*_*CTX-M1-like*_ co-occurred with genes conferring resistance to phenicols (*cmlA* and *catA2*, respectively). Furthermore, *cmlA* showed positive associations with genera *Lactobacillus* and *Subdoligranulum*, which positively correlated with genes conferring resistance to 3GCs (*bla*_*CMY-2*_ and *bla*_*SHV*_, respectively), corroborating the hypothesis of a co-selection for resistance. Although *Lactobacillus* and *Subdoligranulum* are beneficial for the chicken gut health, *cmlA*, *bla*_*CMY-2*_, and *bla*_*SHV*_ genes have been previously described on mobile genetic elements (MGEs) carrying multiple resistance determinants^20–22^, thus contributing to the emergence of multidrug-resistant bacteria, including human and animal pathogens, and representing a concern for veterinary and public health. The molecular detection of ARGs was performed on the total DNA, thus the associations inferred via statistical analysis do not necessarily mean that a taxon harboured a specific resistance gene; however, *cmlA* and *bla* genes have been previously reported in *Lactobacillaceae*^23,24^ and Firmicutes^25^. Similarly, positively correlations between genes conferring resistance to phenicols (i.e. *catA1* and *catB3*) and genera *Ruminococcus* and phylum Firmicutes have been described^26^. The administration of amoxicillin also seems to exert selective pressure on *bla*_*SHV*_ and *bla*_*CMY-2*_; even if transitory, it represents another concern for public health, since these genes conferring resistance to 3GCs are able to be transferred to the soil through fertilization with livestock manure, which is an agricultural common practice, and to spread and persist in the environment^27,28^. Considering the importance regained by chloramphenicol for the treatment of human infections, positive associations between *floR* and *Escherichia/Shigella* and *Enterococcus* represent a threat to human health, because bacteria belonging to these genera can cause severe infections in humans and can harbour multidrug resistance determinants in MEGs, which can be transferred to other bacterial species^5,29–31^.

Caecum samples collected at the slaughterhouse did not show any significant difference in ARGs abundance and microbial community composition between treated and untreated groups. Birds of all groups showed respiratory and intestinal symptoms and therefore were treated with doxycycline for five consecutive days from day 33 onwards. This treatment could be responsible for the flattening of the results, hampering to investigate the long-term effect of the administration of amoxicillin and thiamphenicol on the chicken gut microbiota and associated AMR.

In conclusion, data gathered in the present study suggest that amoxicillin and thiamphenicol treatments might contribute to the co-selection and persistence of resistant bacteria and ARGs against β-lactams (including 3GCs) and phenicols in the chicken gut, posing a health threat. Indeed, conventional poultry farming manure used for soil fertilization can promote the spread and dissemination of AMR in the environment and possibly the transmission to other animals and also humans, via dispersion into waterways or by entering the food chain^28,32^. Therefore, the use of these antimicrobials for the treatment of bacterial infections in poultry farming should be reconsidered, as a reduction in their use could indirectly contribute to reducing the impact of AMR in a One Health perspective.

## Materials and methods

### Animals and samples collection

One hundred one-day-old healthy Ross 308 chicks from a single hatchery were randomly allocated in five different pens in the animal facility of the Department of Veterinary Science, University of Turin, and reared until 46 days of age as part of a zootechnical trial. This trial was approved by the Ethics and Animal Welfare Committee of the Department of Veterinary Sciences (protocol n. 2796/2020), University of Turin (Italy). All broilers were vaccinated against Marek’s disease and Infectious Bursal disease at the hatchery. Additionally, one-day old chicks were vaccinated against coccidiosis (coarse spray), Newcastle disease and Infectious Bronchitis disease (fine spray). Each pen was equipped with a bucket-type feeder and a drinker with wood shavings as litter. The environmental conditions (lighting, temperature, relative humidity, and ventilation) were controlled accordingly to the Ross broiler management guidelines. Birds received the following feeding program to meet the standard nutritional requirements: a commercial starter diet (230 g/kg of CP) from day 0 to 12 (starter period) and a commercial grower diet (185 g/kg of CP) from day 13 to 46 (finisher period). Two groups (AMX1 and AMX2) were administered 20 mg of amoxicillin per kg per live weight per day for three consecutive days, once in the morning and once in the evening with an interval of 12 h, while two groups (THP1 and THP2) were administered with 57 mg of thiamphenicol per kg per live weight per day for three consecutive days. Chicks in the AMX1 and THP1 groups were treated from day 5 onwards, while those in the AMX2 and THP2 groups were treated from day 21 onwards. The control group did not receive any antimicrobial. On day 30 of age, birds of all groups started showing respiratory and intestinal symptoms; therefore, the farm veterinarian prescribed and administered 20 mg of doxycycline per kg of live weight a day for five consecutive days from day 33 onwards. All treatments were administered via drinking water. From each group, six birds were randomly selected and cloacal swabs taken on day 1 (T0). Birds in the AMX1, THP1 and control groups were swabbed on 1 d.p.t. (T1), 12 d.p.t. (T2) and 21 d.p.t. (T3). Cloacal swabs were collected from AMX2, THP2 and control groups on days 1 d.p.t. (T1) and 9 d.p.t. (T2). A total of 120 cloacal swabs were collected. Within one hour after sampling, swabs were placed at – 80 °C until DNA extraction. At the end of the rearing cycle (46 days of age) and after the regular withdrawal period and the complete remission of symptoms, birds were regularly slaughtered and caecum contents were aseptically collected and immediately frozen in liquid nitrogen.

### DNA extraction

Microbial DNA was extracted from the swabs using a commercial kit (QIAamp UCP Pathogen Mini Kit, Qiagen, Germany), and from each swab, two aliquots were prepared to be used in the downstream analyses. Meanwhile, DNA was extracted from 250 mg of caecal content using the DNeasy PowerSoil DNA Isolation Kit (Qiagen, Hilden, Germany), following manufacturer’s recommendations. DNA quality and quantity were assessed using the Qubit 2.0 Fluorometer (Thermo Fisher Scientific, Monza, Italy).

### 16S rRNA gene sequencing and data analysis

NGS-based 16S rRNA was performed to identify and compare bacteria present in each sample. Briefly, the V3-V4 region of the 16S rRNA gene was amplified with primers 341F/R806 with overhangs adapters sequences^33^, and the second stage PCR was performed to add the IDT for Illumina Nextera DNA UD Indexes (IDT). All PCRs were carried out using KAPA HiFi HotStart ReadyMix (Roche) in a 2720 thermal cycler (Applied Biosystems, Waltham, MA), applying the amplification protocol 95 °C for 3 min (min), 25 or 8 cycles at 95 °C for 30 s (s), 55 °C for 30 s and 72 °C for 30 s, for the first and the second stage PCR, respectively. After both PCR stages, amplicons were purified using the SPRIselect purification kit (Beckman Coulter, Brea, CA). Libraries were quantified using Qubit 2.0 Fluorometer (Thermo Fisher Scientific), pooled and sequenced using the Illumina MiSeq sequencing platform (San Diego, California, USA) with a 2 × 300 bp paired-end approach. Within the Quantitative Insights into Microbial Ecology 2 (QIIME2 version 2019.4) software, the DADA2 package was used for 16S rRNA data analysis^34,35^. Taxa assignment was carried out using SILVA-Naive Bayes sklearn trained database^36^, while the on-line software Calypso (http://cgenome.net/wiki/index.php/Calypso) and Galaxy (https://galaxyproject.org/)^37^ were used for the statistical analysis of microbial communities. The microbial community composition was explored using heatmaps, while microbial diversity within each group (α-diversity) was expressed using Shannon and Simpson indexes. Differences in microbial composition among groups (β-diversity) were assessed using permutational multivariable analysis of variance (PERMANOVA) based on the Bray–Curtis dissimilar measure using the Adonis function and visualized with non-metric multidimensional scaling (NMDS). The linear discriminant analysis (LDA) effect size method (LEfSe) was used to identify taxa most likely to explain differences between groups at the same time point.

### Quantitative PCR (qPCR) analysis of ARGs

Extracted DNA was screened by qPCR to detect and quantify 14 ARGs conferring resistance to β-lactams (i.e. *bla*_*TEM-1*_*, bla*_*SHV*_*, bla*_*CTX-M-1like*_*, bla*_*CMY-2*_*, bla*_*OXA-1*_*, bla*_*OXA-48*_*, bla*_*VIM-2*_ and *bla*_*NDM*_) and phenicols (i.e. *catA1, catA2, catA3, catB* Group3, *floR* and *cmlA*). Primers’ sequences, optimal concentrations, annealing and melting temperatures, and positive controls used are reported in Supplementary Table 1. All samples were tested for each gene in triplicate using PowerUp™ SYBR Green Master Mix (Thermo Fisher Scientific) with an optimal concentration of each primer in a LightCycler480 Roche (Roche, Basel, Switzerland) real-time platform and the mean values (copy number of each ARG) were estimated. However, since the absolute abundance of ARGs in a given sample is not a significant value, as it is proportional to the total DNA present in the sample, ARGs relative abundance was calculated by normalizing the ARG copies number to 16S rRNA gene copies, and used in the statistical analysis.

### Statistical analysis

Differences in α-diversity or ARG abundance among groups (e.g. AMX1, THP1 and control) at the same time points (e.g. T0–T3) were tested for significance using generalized linear models (GLMs) with a log link and Gaussian error distribution (log-transformed Shannon index values) or gamma error distribution (ARG abundances). Only ARGs with ≥ 10% prevalence over all samples were included in the analysis. Hierarchical clustering was used to assess associations between presence/absence of each ARG and groups and time points. Differences in relative abundance of microbial taxa among the three groups were tested for significance using multivariate regression analysis with several dependent variables (i.e. log-transformed relative abundances of the microbial taxa). The same approach was used to assess associations between the relative abundance of the different taxa and ARG abundance. Also for these analyses, only taxa and ARGs present at a confident level of detection of 10% prevalence over samples were included. Furthermore, Spearman’s rank correlation was used to assess the co-occurrence of ARGs conferring resistance to β-lactams and phenicols. All models included cluster-robust (Sandwich) variance estimators to account for clustering of samples originating from the same chicks over time points. Statistical significance was set at p < 0.05. Statistical analysis and data visualization were carried out in R (version 4.2.1) (https://www.r-project.org/) using package Stats (version 4.1.2), Stata 16 (StataCorp, USA) and GraphPad Prism version 9.2.0 (https://www.graphpad.com).

### Ethical statement

The zootechnical trial was approved by Ethics and Animal Welfare Committee of the Department of Veterinary Sciences (protocol n. 2796/2020), University of Turin (Italy). (https://www.veterinaria.unito.it/do/organi.pl/Show?_id=twsn). All international, national and/or institutional guidelines for care and use of animals were followed. The authors declare that the animal results of the study are reported in accordance with ARRIVE guidelines (https://arriveguidelines.org).

## Supplementary Information

Supplementary Figure S1.

Supplementary Figure S2.

Supplementary Figure S3.

Supplementary Figure S4.

Supplementary Figure S5.

Supplementary Table 1.

## Data Availability

The datasets generated and/or analysed during the current study are available in the NCBI repository, https://www.ncbi.nlm.nih.gov/bioproject/PRJNA872901, BioProject ID PRJNA872901.
