# Supplementary material for: Amoxicillin and thiamphenicol treatments may influence the co-selection of resistance genes in the chicken gut microbiota
Source: Sci Rep. 2022 Nov 27;12:20413. doi: 10.1038/s41598-022-24927-7 (PMC9701756; doi:10.1038/s41598-022-24927-7)
Supplement: Supplementary file 1 — Supplementary Figure S1. [file 41598_2022_24927_MOESM1_ESM.pptx]

## Slide 1
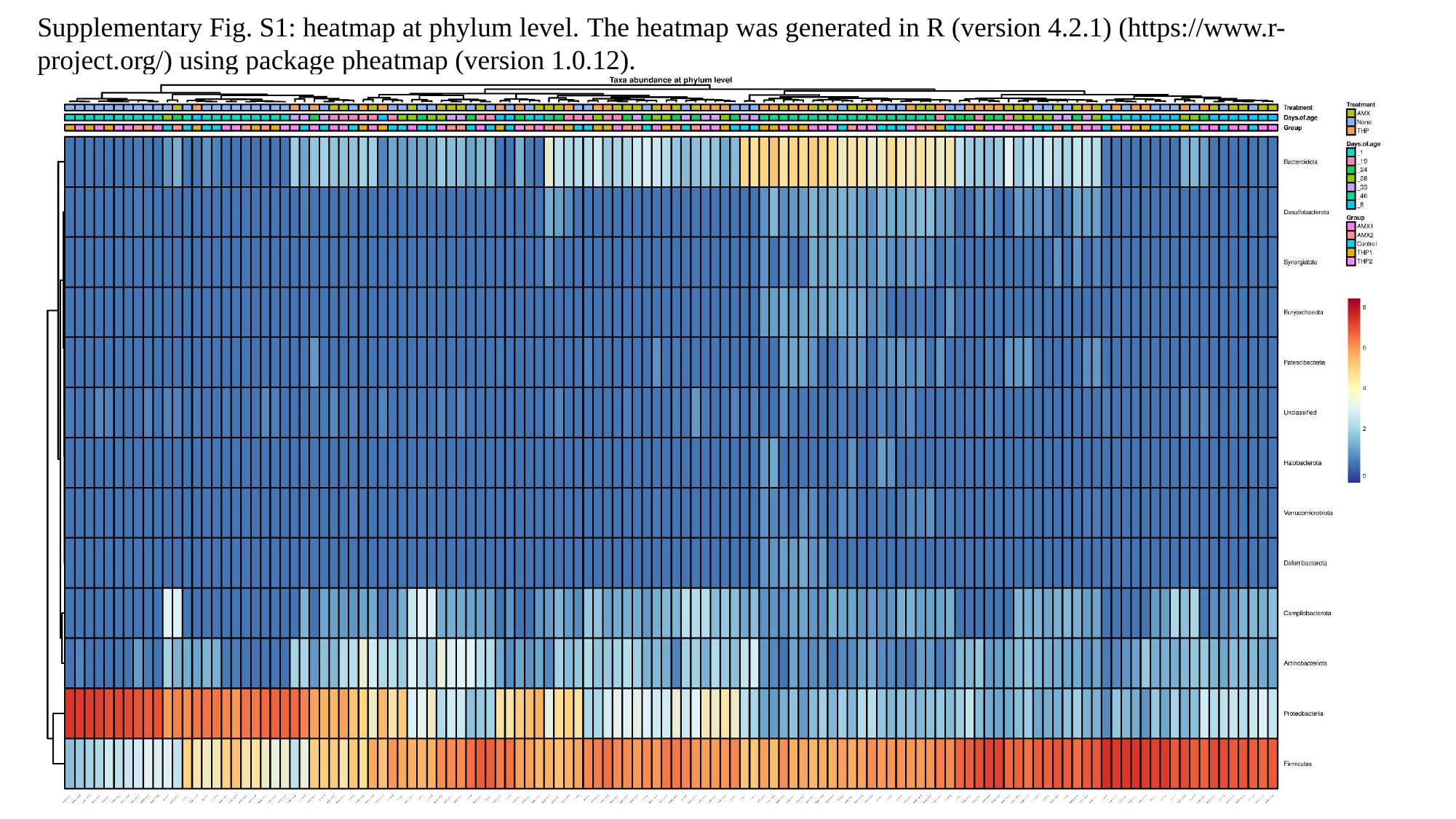

Supplementary Fig. S1: heatmap at phylum level. The heatmap was generated in R (version 4.2.1) (https://www.r-project.org/) using package pheatmap (version 1.0.12).
