# Supplementary figures and images for: Amoxicillin and thiamphenicol treatments may influence the co-selection of resistance genes in the chicken gut microbiota
Source: Sci Rep. 2022 Nov 27;12:20413. doi: 10.1038/s41598-022-24927-7 (PMC9701756; doi:10.1038/s41598-022-24927-7)

## Slide 1
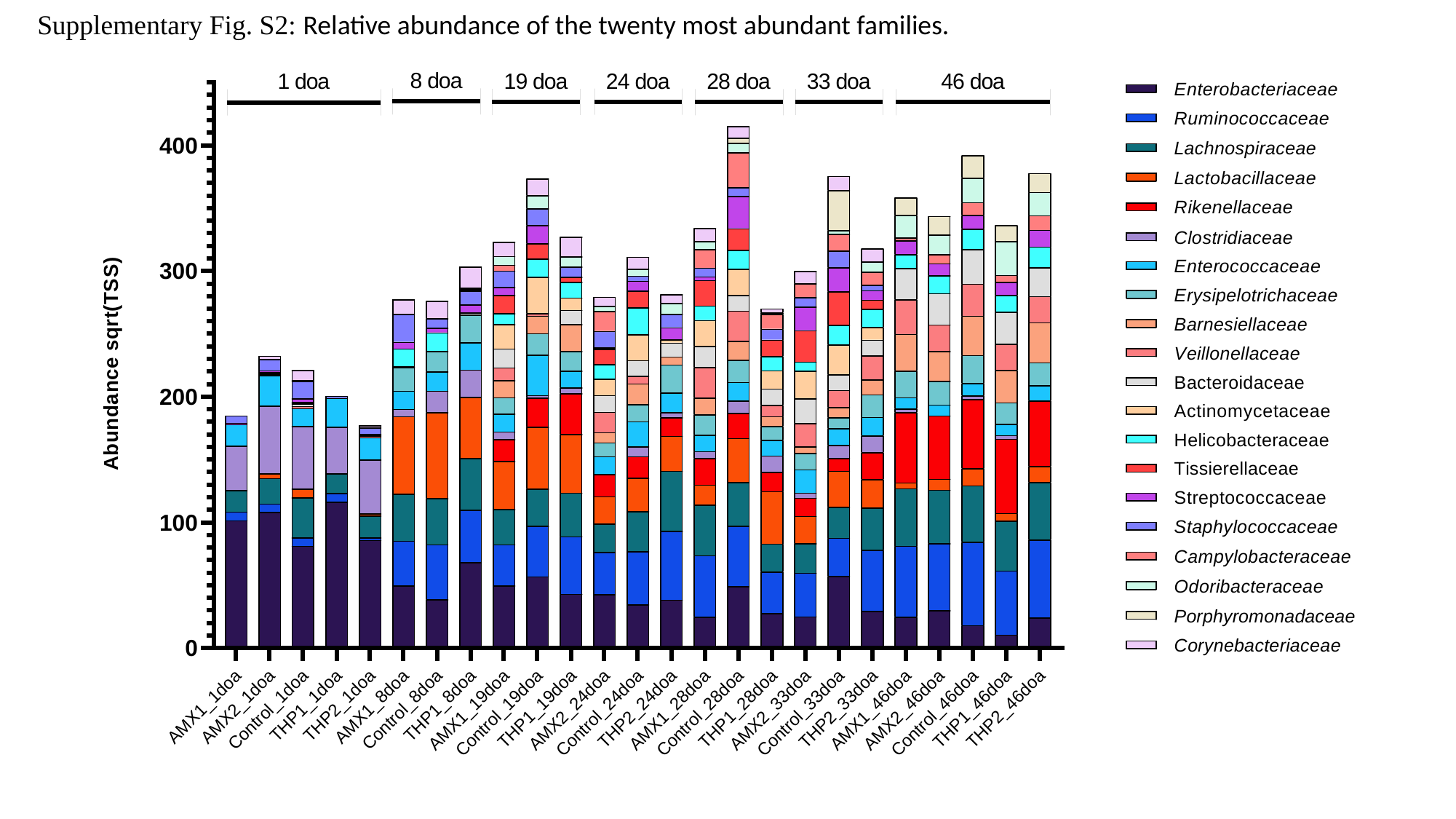

Supplementary Fig. S2: Relative abundance of the twenty most abundant families.

Supplement: Supplementary file 2 — Supplementary Figure S2. [file 41598_2022_24927_MOESM2_ESM.pptx]
