# Supplementary material for: Amoxicillin and thiamphenicol treatments may influence the co-selection of resistance genes in the chicken gut microbiota
Source: Sci Rep. 2022 Nov 27;12:20413. doi: 10.1038/s41598-022-24927-7 (PMC9701756; doi:10.1038/s41598-022-24927-7)
Supplement: Supplementary file 3 — Supplementary Figure S3. [file 41598_2022_24927_MOESM3_ESM.pptx]

## Slide 1
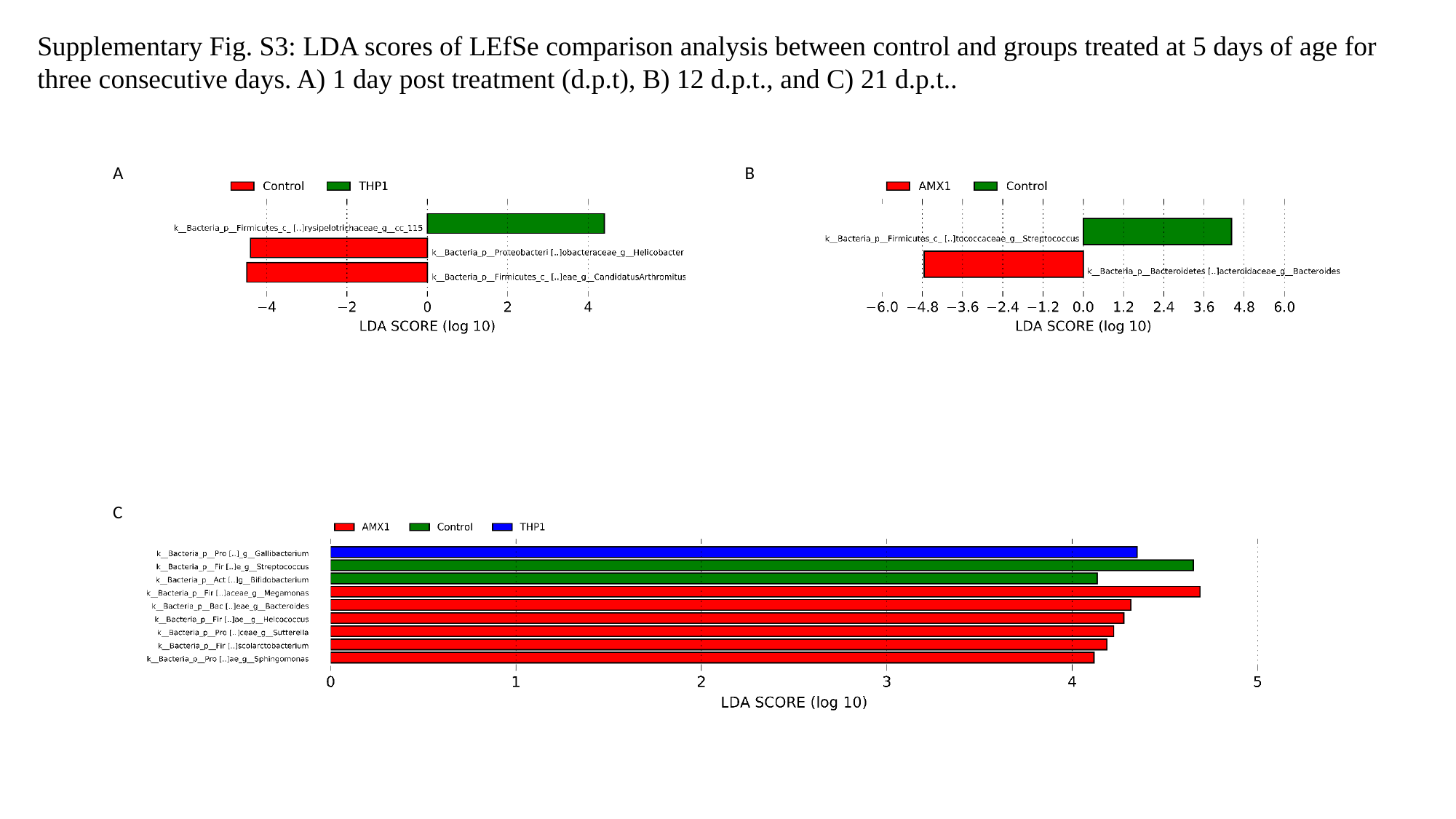

Supplementary Fig. S3: LDA scores of LEfSe comparison analysis between control and groups treated at 5 days of age for three consecutive days. A) 1 day post treatment (d.p.t), B) 12 d.p.t., and C) 21 d.p.t..
B
A
C
