# Supplementary material for: Amoxicillin and thiamphenicol treatments may influence the co-selection of resistance genes in the chicken gut microbiota
Source: Sci Rep. 2022 Nov 27;12:20413. doi: 10.1038/s41598-022-24927-7 (PMC9701756; doi:10.1038/s41598-022-24927-7)
Supplement: Supplementary file 4 — Supplementary Figure S4. [file 41598_2022_24927_MOESM4_ESM.pptx]

## Slide 1
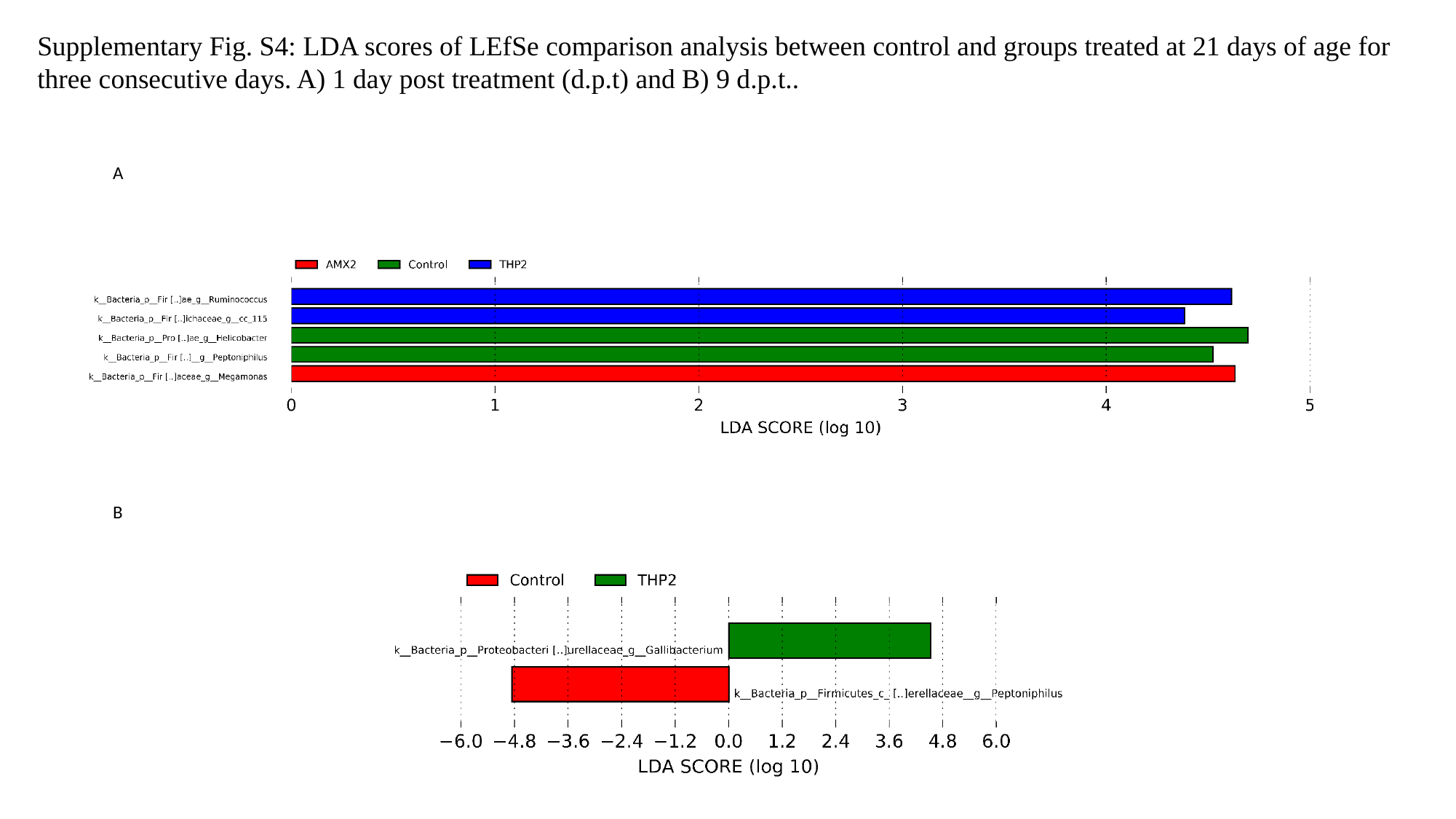

Supplementary Fig. S4: LDA scores of LEfSe comparison analysis between control and groups treated at 21 days of age for three consecutive days. A) 1 day post treatment (d.p.t) and B) 9 d.p.t..
A
B
