# Supplementary material for: Amoxicillin and thiamphenicol treatments may influence the co-selection of resistance genes in the chicken gut microbiota
Source: Sci Rep. 2022 Nov 27;12:20413. doi: 10.1038/s41598-022-24927-7 (PMC9701756; doi:10.1038/s41598-022-24927-7)
Supplement: Supplementary file 5 — Supplementary Figure S5. [file 41598_2022_24927_MOESM5_ESM.pptx]

## Slide 1
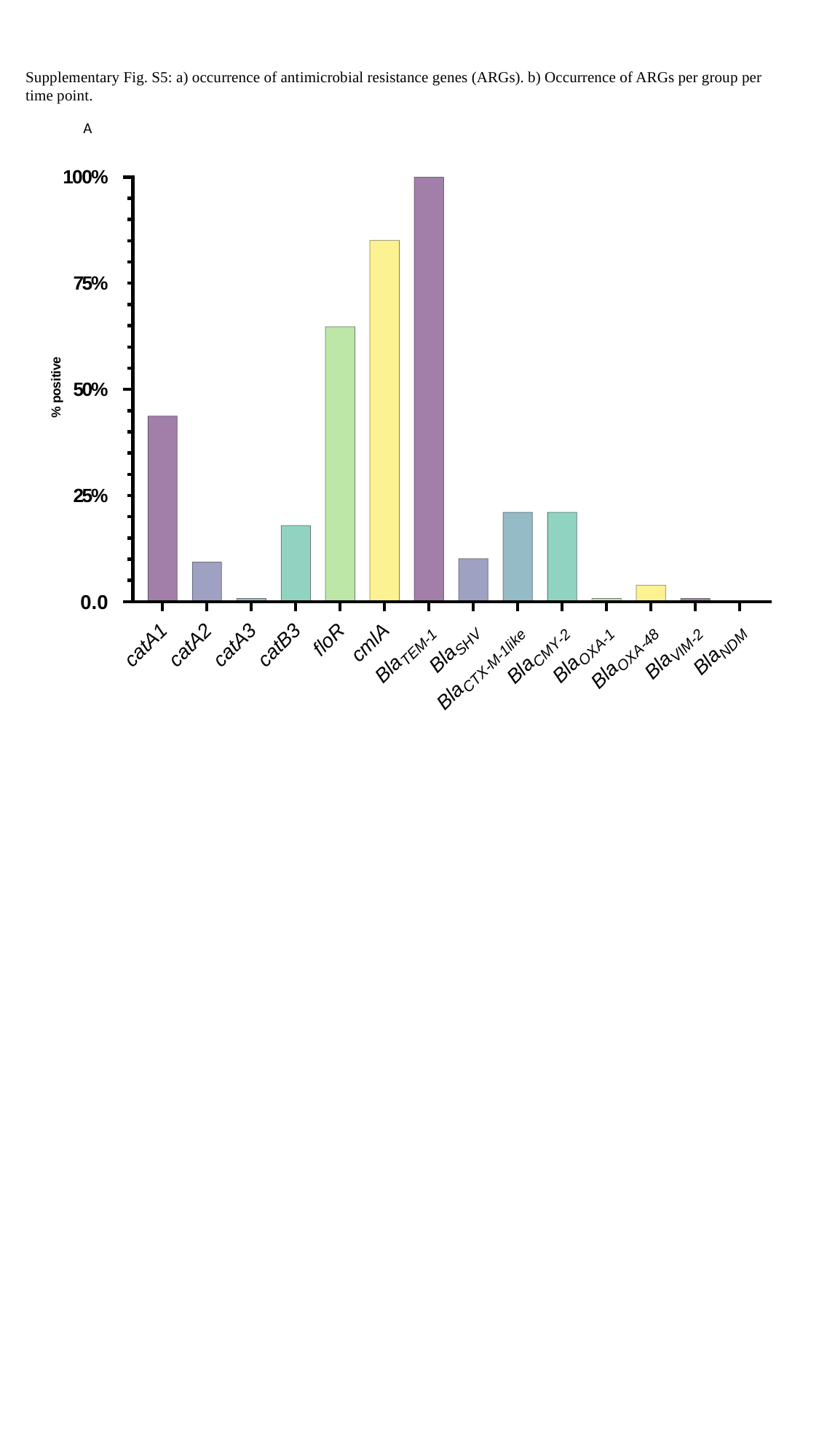

Supplementary Fig. S5: a) occurrence of antimicrobial resistance genes (ARGs). b) Occurrence of ARGs per group per time point.
A

## Slide 2
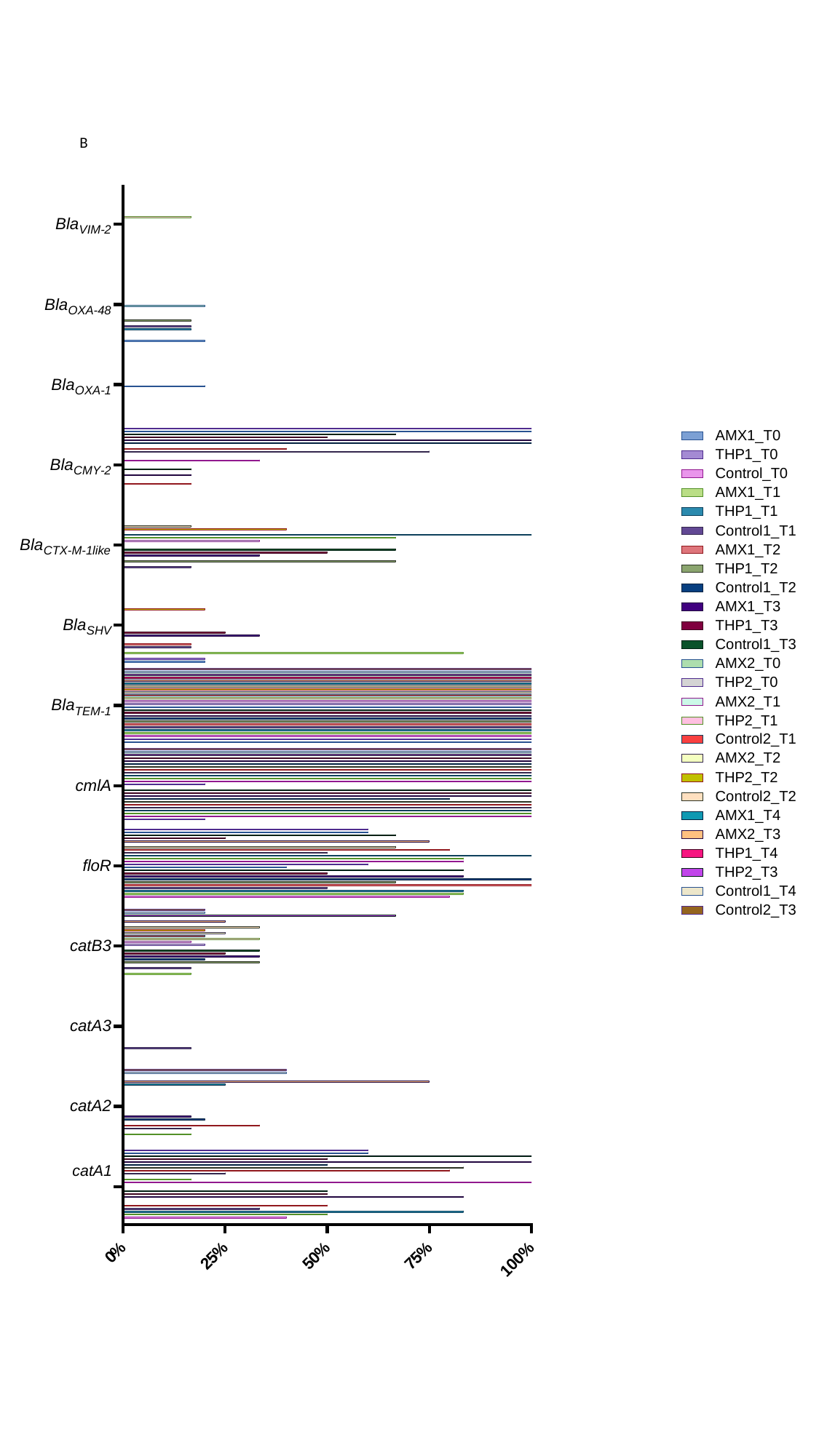

B
